# The baseline comorbidity burden affects survival in elderly patients with acute myeloid leukemia receiving hypomethylating agents: Results from a multicentric clinical study

**DOI:** 10.1002/cam4.5858

**Published:** 2023-03-31

**Authors:** Giovanni Marconi, Anna Candoni, Roberta Di Nicola, Chiara Sartor, Sarah Parisi, Mariachiara Abbenante, Jacopo Nanni, Gianluca Cristiano, Letizia Zannoni, Davide Lazzarotto, Benedetta Giannini, Carmen Baldazzi, Lorenza Bandini, Emanuela Ottaviani, Nicoletta Testoni, Chiara Di Giovanni Bezzi, Rania Abd‐alatif, Giulia Ciotti, Renato Fanin, Giovanni Martinelli, Stefania Paolini, Paolo Ricci, Michele Cavo, Cristina Papayannidis, Antonio Curti

**Affiliations:** ^1^ IRCCS Istituto Scientifico Romagnolo per lo Studio e la Cura dei Tumori “Dino Amadori” (IRST) Meldola Italy; ^2^ Department of Experimental, Diagnostic and Specialty Medicine University of Bologna Bologna Italy; ^3^ Division of Hematology University Hospital‐ASUFC Udine Italy; ^4^ Section of Haematology, Department of Medical and Surgical Sciences University of Modena and Reggio Emilia Modena Italy; ^5^ Department of Haematology and Stem Cell Transplantation Unit IRCCS “Casa Sollievo della Sofferenza” Hospital San Giovanni Rotondo Italy; ^6^ IRCCS Azienda Ospedaliero‐Universitaria di Bologna Istituto di Ematologia “Seràgnoli” Bologna Italy; ^7^ Assistenza Domiciliare Ail Bologna Bologna Italy; ^8^ Onco Hematology, Department of Oncology–Veneto Institute of Oncology IOV–IRCCS Padua Italy

**Keywords:** acute myeloid leukemia, elderly, fitness, hypomethylating agents, prognosis

## Abstract

**Background:**

In older patients with acute myeloid leukemia (AML), the definition of fitness, prognosis, and risk of death represents an open question.

**Methods:**

In the present study, we tested the impact on survival of disease‐ and patient‐related parameters in a large cohort of elderly AML patients homogeneously assigned to treatment with hypomethylating agents (HMAs).

**Results:**

In 131 patients with a median age of 76 years, we confirmed that early response (<0.001) and biology‐based risk classification (*p* = 0.003) can select patients with better‐predicted survival. However, a full disease‐oriented model had limitations in stratifying our patients, prompting us to investigate the impact of baseline comorbidities on overall survival basing on a comorbidity score. The albumin level (*p* = 0.001) and the presence of lung disease (*p* = 0.013) had a single‐variable impact on prognosis. The baseline comorbidity burden was a powerful predictor of patients' frailty, correlating with increased incidence of adverse events, especially infections, and predicted overall survival (*p* < 0.001).

**Conclusion:**

The comorbidity burden may contribute to impact prognosis in addition to disease biology. While the therapeutic armamentarium of elderly AML is improving, a comprehensive approach that combines AML biology with tailored interventions to patients' frailty is likely to fully exploit the anti‐leukemia potential of novel drugs.

## Graphical abstract

In this study, we tested the impact on survival of disease‐ and patient‐related parameters in a large cohort of elderly AML patients assigned to treatment with HMAs.

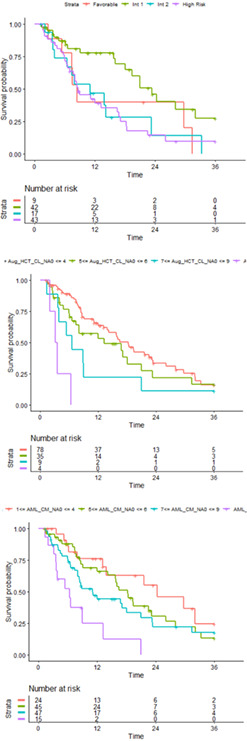

## 1 INTRODUCTION

Despite recent advances, the 5‐year patients' overall survival (OS) of acute myeloid leukemia (AML) is still largely unsatisfactory, reaching only 30% and dropping to 5%–10% in the elderly.^1^, ^2^, ^3^ Older patients represent an unfavorable subset for whom an optimization of the clinical management is far‐reaching and crucial questions are still open and unresolved. Of note, several effective treatment regimens have recently entered the clinical stage, thus urging the need for better stratification of the elderly based on both disease‐related factors and features capturing patient's characteristics.^4^, ^5^, ^6^ Among the latter, the comorbidity burden is emerging as a crucial point, but its impact on survival and its value as a predictor of global outcome has not been fully elucidated.

In the last years, many efforts have been made to assess comorbidities as part of the general process of fitness definition, which guides the decision to treat older AML patients with intensive chemotherapy or less intensive therapies.^7^, ^8^, ^9^, ^10^, ^11^ A comprehensive geriatric assessment, which takes into consideration multiple domains (social, physical, cognitive, and clinical)^11^, ^12^, ^13^ have been evaluated as an innovative prognostic tool in elderly AML, but its poor scalability and a general lack of consensus on the parameters to include have limited its impact on clinical practice.^14^ To capture the comorbidity burden and define patient's fitness several additional and simplified tools have been proposed.^11^, ^12^, ^13^, ^15^, ^16^, ^17^, ^18^ In a seminal work, Sorror et al demonstrated the prognostic power of extended comorbidity index in patients undergoing transplant.^19^ More recently, such score has been implemented in the AML setting (augmented‐comorbidity index, A_CI) and integrated with AML‐related factors, such as cytogenetic‐molecular status (AML—composite model, AML‐CM).^20^, ^21^ Although the use of these scores has certainly improved the capacity of dissecting patients population, the prognostic significance of comorbidities and their correlation with AML biology in patients homogeneously treated with non‐intensive therapies, especially in a real‐life setting, is still an area of investigation.

In the present study, we defined the overlapping influences of disease biology, age, and non‐AML related factors as captured by AML‐CM on the prognosis of a large set of elderly patients, who had been considered by physician's choice unfit for intensive chemotherapy and were, then, treated with hypomethylating agents (HMAs).

## 2 METHODS

### 2.1 Patients

We collected data from consecutive patients with AML treated at the Institute of Hematology L. and A. Serágnoli—Policlinico S. Orsola‐Malpighi in Bologna, at Division of Hematology in University Hospital‐ASUFC in Udine, and Istituto Romagnolo per lo Studio dei Tumori “Dino Amadori”—IRST in Meldola from 1st Dec 2010 to 1st Jun 2021. Informed consent was obtained from every patient at the time of diagnosis in accordance with GCP and as approved by the local ethical review board. Within the entire patient population, we report data on elderly patients who received front‐line, single‐agent HMAs. Major criteria for enrolment were old age (>65 years old), AML defined according to WHO 2016 criteria excluding acute promyelocytic leukemia,^22^ and front‐line therapy with HMAs. Exclusion criteria were to be enrolled at diagnosis in studies with investigational agents and to have received frontline chemotherapy (excluding HU and cytarabine cytoreduction). Patients selected for this report received at least one dose of azacytidine or decitabine after AML diagnosis.

### 2.2 Data collection

For every patient, we collected data on the onset of the disease, including age, history of the antecedent hematologic disorder, history of antecedent diseases, baseline comorbidities, clinical and laboratory features, and cytogenetic and molecular characteristics. Survival, disease status, therapies for AML, and adverse events (AE) were collected for the entire duration of follow‐up. For analysis purposes, we considered AEs, severe AEs, infections, and severe infections as AE categories.

### 2.3 Definitions

AML was diagnosed and categorized according to WHO2008 or WHO2016 criteria and classified in the study database according to WHO2016 criteria.^22^ Response and relapse were defined according to 2017 ELN recommendations^23^; hematological improvement was defined as the loss of transfusion dependence lasting at least 56 days. Disease status was assessed after courses 2, 4, 6, and every 6 courses thereafter in patients who changed their hematological condition from baseline. Overall survival (OS) was defined as the time from diagnosis to death for any cause. AEs were defined according to CTCAE 5.0.^24^

### 2.4 Cytogenetics, molecular analyses, and risk stratification

Review of cytogenetic status included karyotype based on a minimum of 20 metaphase cells. Molecular analyses were performed on DNA or RNA of mononuclear cells by polymerase chain reaction, or Sanger sequencing, or capillary electrophoresis, as appropriate. The cytogenetic and molecular risk was stratified according to ELN 2010 Prognostic System.^25^ Due to the lack of advanced molecular biology (TP53, ASXL1, CEBPA, RUNX1, FLT3 allelic ratio) in most of the cases, ELN2017 risk stratification did not apply to the population.

### 2.5 Comorbidities

Comorbidities were defined as previously reported by Mukherjee and colleagues^20^; For the univariate analysis, the presence or absence of comorbidity was used instead of the intensity score, to manage the low prevalence. Augmented‐comorbidity index (A‐CI) and AML‐composite model (AML‐CM) were calculated as previously defined and prognostic groups were defined accordingly, including cytogenetic/molecular risk.^20^ For the creation of the scores, we included patients for which more than 80% of variables were known; non‐available values were considered equal to nil, to guarantee the most conservative approach.

### 2.6 Therapy regimens

Azacytidine was administered subcutaneously at the dose of 75 mg/sqm on days 1–5, 8–9, or 1–7 of consecutive 28‐day courses. Decitabine was administered intravenously at the dose of 20 mg/sqm on days 1–5 of consecutive 28‐day courses. Courses delays and dose reductions were managed according to standard clinical practice to allow recovery from hematology toxicities in patients who are in complete remission or to consent to lower the grade of ongoing adverse events. Dose delays of more than 2 weeks not due to adverse events were registered as treatment interruption.

### 2.7 Statistical analysis

Statistical analyses were performed on the entire sample population, if not differently specified. Data collected have been summarized by descriptive statistics such as mean and standard deviation or median with its interquartile range (IQR) for continuously distributed variables, otherwise, rates and relative frequencies have been reported for categorical distributions. Fisher exact test or Chi‐squared test have been adopted to evaluate associations between groups and nominal variables were appropriate. The Mann–Whitney test has been applied, alternatively, to assess differences between medians in non‐normal distributions. Survival analysis has been performed by means of the Kaplan–Meier estimator and its algorithm for survival curve generation. Subgroup analyses were conducted whenever appropriate. Log‐rank test was used to compare survivals curves probabilities, while semiparametric Cox regression analysis has been adopted to estimate Hazard Ratios (HR). All the analyses were conducted using R language and environment for statistical computing (R Foundation for Statistical Computing). 0.05 was taken as the cut‐off for two‐sided *p*‐values statistical significance. All confidence intervals (CI) were reported as 95% CI.

## 3 RESULTS

### 3.1 Patient population

In total, 131 elderly patients received 1st line HMAs at the participating institutions. Baseline characteristics are reported in Table 1. In our patient set, the median age was 76 years (IQR 72–79). Seventy‐seven out of 131 patients (58.8%) had de novo AML, 32/131 (32.8%) had secondary AML, and 11/131 (8.4%) had therapy‐related AML. Out of 123 patients with evaluable cytogenetics, 43 (34.9%) had complex karyotype, 1 (0.8%) inv (16), 59 (48.4%) normal karyotype, 18 (14.7%) other alterations; 8/108 patients harbored FLT3 ITD mutation (7.4%, 23 not tested), 12/101 NPM1 mutation (11.9%, 30 not tested). Based on these data, 111 patients were evaluable for ELN 2010 risk stratification; 9 out of 111 patients (8.1%) were stratified in the low risk, 42/111 (37.8%) in intermediate‐1 risk, 17/111 (15.3%) in intermediate‐2 risk, and 43/111 (38.7%) in high‐risk class.

**TABLE 1:** Patients' characteristics.

Characteristics at AML diagnosis	*N* patients = 131
Age at diagnosis	
Median, years (IQR)	76 (72–79)
AML type, *N* (%)	
de novo	77 (58.8%)
Onset after MDS	33 (25.2%)
Onset after PMF/TE/PV/other chronic myeloid diseases	10 (7.6%)
Therapy‐related	11 (8.4%)
FLT3‐ITD *N*/patients in which mutation was searched (%)	8/108 (7.4%)
NPM1 mutated *N*/patients in which mutation was searched (%)	12/101 (11.9%)
Karyotype analysis, *N* (%)	
Complex	32 (26.2%)
del (17)	1 (0.8%)
del (5)	4 (3.3%)
del (7)	5 (4.1%)
inv (3)	2 (1.6%)
inv (16)	1 (0.8%)
MLL rearrangement	1 (0.8%)
Normal	59 (48.4%)
Other	17 (13.9%)
Risk stratification system ELN2010, *N* (%)	
Favorable	9 (8.1%)
Intermediate I	43 (38.7%)
Intermediate II	42 (37.8%)
Adverse	17 (15.3%)
Treatment, *N* (%)	
Azacitidine	57 (43.5%)
Decitabine	74 (56.5%)

Abbreviations: AML, acute myeloid leukemia; IQR, interquartile range; MDS, myelodysplastic syndrome; PMF, primary myelofibrosis; TE, essential thrombocythemia; PV, polycythemia vera.

As expected, most of the patients had at least one comorbidity (Table 2). Particularly, baseline cardiovascular comorbidity was present in 20/130 (15.4%, 1 no details), arrhythmia in 29/130 (22.1%, 1 no details), cerebrovascular comorbidity in 11/131 (8.4%), diabetes in 20/131 (15.3%), obesity 18/129 (14%, 2 no data), kidney disease 15/130 (11.5%, 1 no details), lung chronic disease 19/130 (14.6% 1 no details), hypoalbuminemia in 25/111 patients (22.5%, 20 no details).

**TABLE 2:** Distribution of baseline comorbidities in our patient set; comorbidities were defined according to Mukherjee et al., Jama Oncol. 2017.^20^

Baseline comorbidity	Patients affected, *N* (%)
Elevated LDH	86 (78.2%)
Prior tumor	32 (24.4%)
Arrhythmia	29 (22.1%)
Hypoalbuminemia	25 (22.5%)
Thrombocytopenia	22 (16.9%)
Diabetes	20 (15.3%)
Cardiovascular comorbidities	20 (15.4%)
Lung disease	19 (14.6%)
Ongoing infections	18 (13.7%)
Obesity	18 (14.0%)
Kidney disease	15 (11.5%)
Rheumatology disease	14 (10.7%)
Cerebrovascular disease	11 (8.4%)
Hepatic disease	9 (7.0%)
Peptic Ulcer	5 (3.8%)
Heart valve disease	3 (2.3%)
IBD	2 (1.5%)
Psychiatric disturbance	(0.8%)

Abbreviations: IBD, inflammatory bowel disorder; LDH, lactic dehydrogenase.

### 3.2 Survival

**FIGURE 1:**
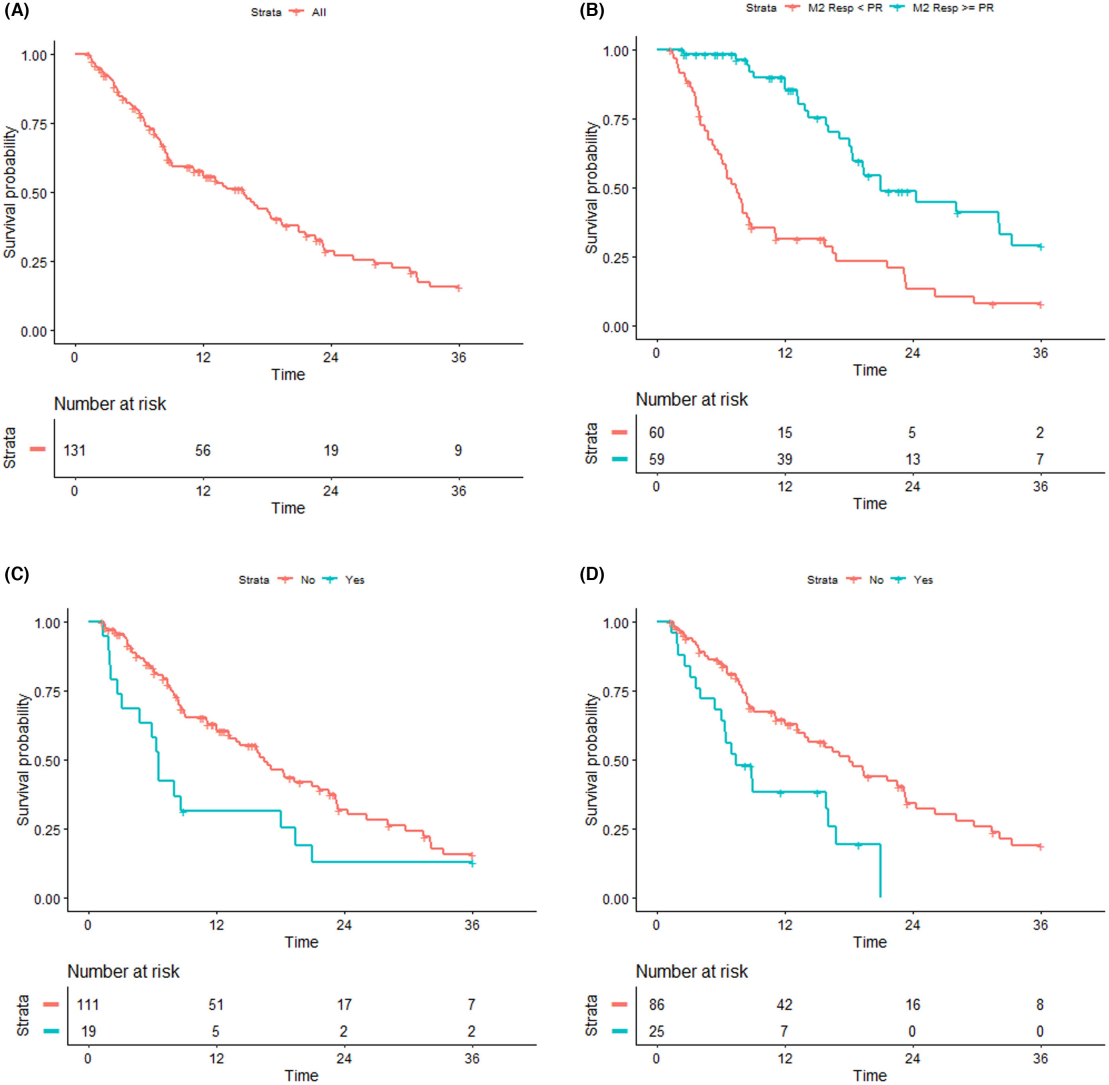
(A) Overall survival in our set; (B) overall survival in patients who obtained CR, CRi, HI, or PR at month 2 compared with patients in stable disease (*p* < 0.001); (C) overall survival in patients with lung disease compared with patients without lung disease (*p* = 0.013) (D) overall survival in patients with low serum albumin compared with patients with normal serum albumin (*p* < 0.001).

With a median follow‐up of 28.2 months (Figure 1A, IQR 11.7–74.3), the median OS of the entire cohort was 15.8 months (95% C.I. 11.2–19.4). Six‐month, 12‐month, and 24‐month survival probability were 79.0% (95% CI 72.1–86.5), 55.4% (95% CI 47.0–65.4), and 28.5% (95% CI 20.4–39.7), respectively.

The type of HMAs used (decitabine versus azacitidine) or age (more or less than 70 years old) had no significant impact on survival.

At month 2, 59 out of 119 patients reached response (49%), including 10 patients with CR, 1 patient with CRi, and 48 patients with partial response. As expected, early response to HMA therapy was highly predictive of better survival (Figure 1B, median OS of 21 months for responders vs 7.4 months for non‐responders, *p* < 0.001). Improved survival was confirmed also for patients who were in response at 4 or 6 months (Figure S1) To evaluate parameters associated with response, prior tumor (*p* = 0.001) and high ELN2010 risk (*p* = 0.003) were associated with a low probability of response.

### 3.3 ELN 2010 risk stratification and baseline comorbidities contribute to defining the probability of survival

**FIGURE 2:**
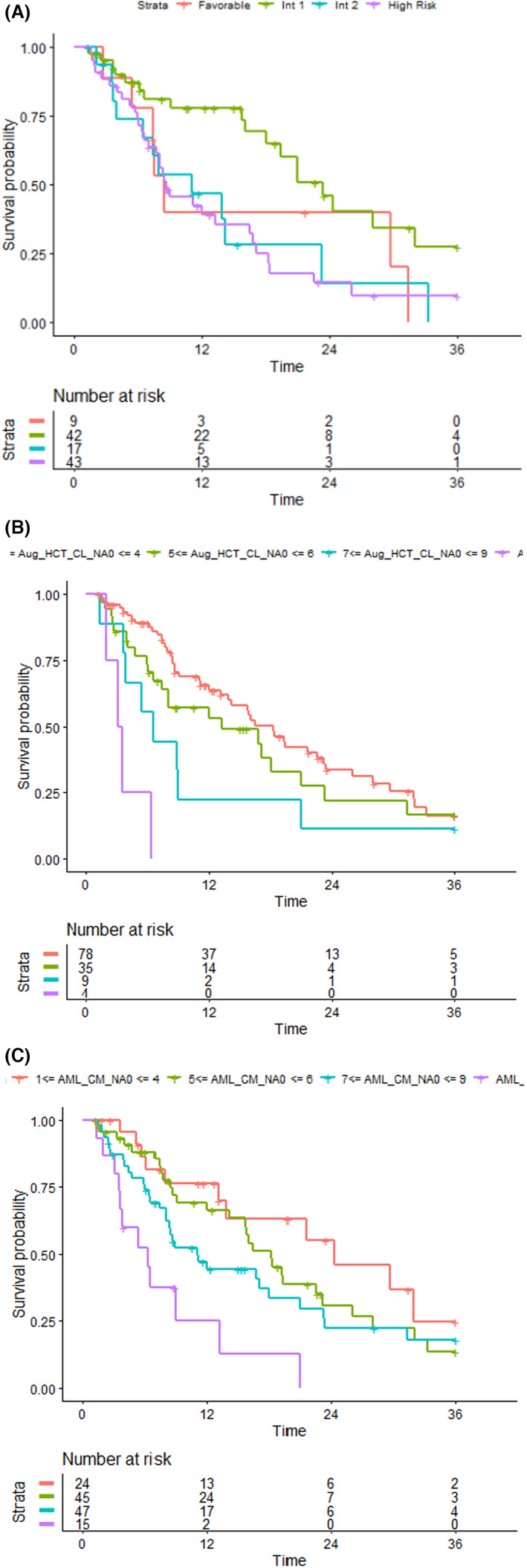
(A) Overall survival according to ELN2010 risk score; (B) overall survival according to augmented A‐CI; (C) overall survival according to AML‐CM score.

Given the great heterogeneity of outcomes in our cohort of patients, we sought to investigate the differential impact on the prognosis of disease‐ and patient‐specific factors. To this aim, molecular and cytogenetic parameters, captured and integrated into ELN2010 risk classification, as well as well‐established comorbidities were specifically correlated with OS. According to ELN2010 risk classification, patients belonging to the int‐1 risk category had the best OS (23.4 months), whereas those in the high‐risk category showed very short OS (6.5 months). Of note, “intermediate‐2” risk patients had a similar, statistically non‐different outcome as high‐risk patients, suggesting that disease‐specific biologic factors, captured by ELN2010 risk classification, might not be fully competent in stratifying our group of patients (Figure 2A). In particular, the median OS of “favorable”, “intermediate‐1”, “intermediate‐2” and “high” risk categories was 8.4, 23.4, 11.1, and 6.5 months, respectively (int‐1 vs high‐risk HR: 0.38 95%, 0.21–0.68; *p* = 0.001). We, then, analyzed the prognostic impact of comorbidities. For each baseline comorbidity, we tested the impact on overall survival. Interestingly, lung disease (Figure 1C, median OS 6.6 months in affected vs. 16.5 months in non‐affected, *p* = 0.013) and hypoalbuminemia (Figure 1D, median OS 7.4 months in affected vs 18 months in non‐affected *p* < 0.001) conferred significantly diminished OS. Taken together, these data may indicate that for better stratification of elderly patients treated with HMAs, disease‐related factors, as captured by ELN risk classification, would be more successfully integrated with the impact of baseline comorbidities.

### 3.4 Augmented‐CI and AML‐CM risk impact on prognosis

The sample size in this study was not sufficient to constitute a new prognostic model. Then, for further analyses, the impact on survival of comorbidities alone and combined with disease‐related factors were assessed by using augmented‐CI (A‐CI) and AML‐CM scores, respectively.^20^ Using A‐CI, OS was negatively associated with comorbidity burden. The median OS was 18.3 months (95% C.I. 13.9–23.4) for patients with low‐baseline comorbidity burden (A‐CI 1–4, *n* = 78) whereas patients with medium (A‐CI 5–6, *n* = 35), high (A‐CI 7–9, *n* = 9), or very high (A‐CI >9, *n* = 4) comorbidity burden had a median OS of 13.2 (95% C.I. 7.4–23.3), 6.6 (95% C.I. 3.9‐NR), and 3.3 (95% C.I. 3.3–NR) months, respectively (Figure 2B, *p* for trend <0.001).

**FIGURE 3:**
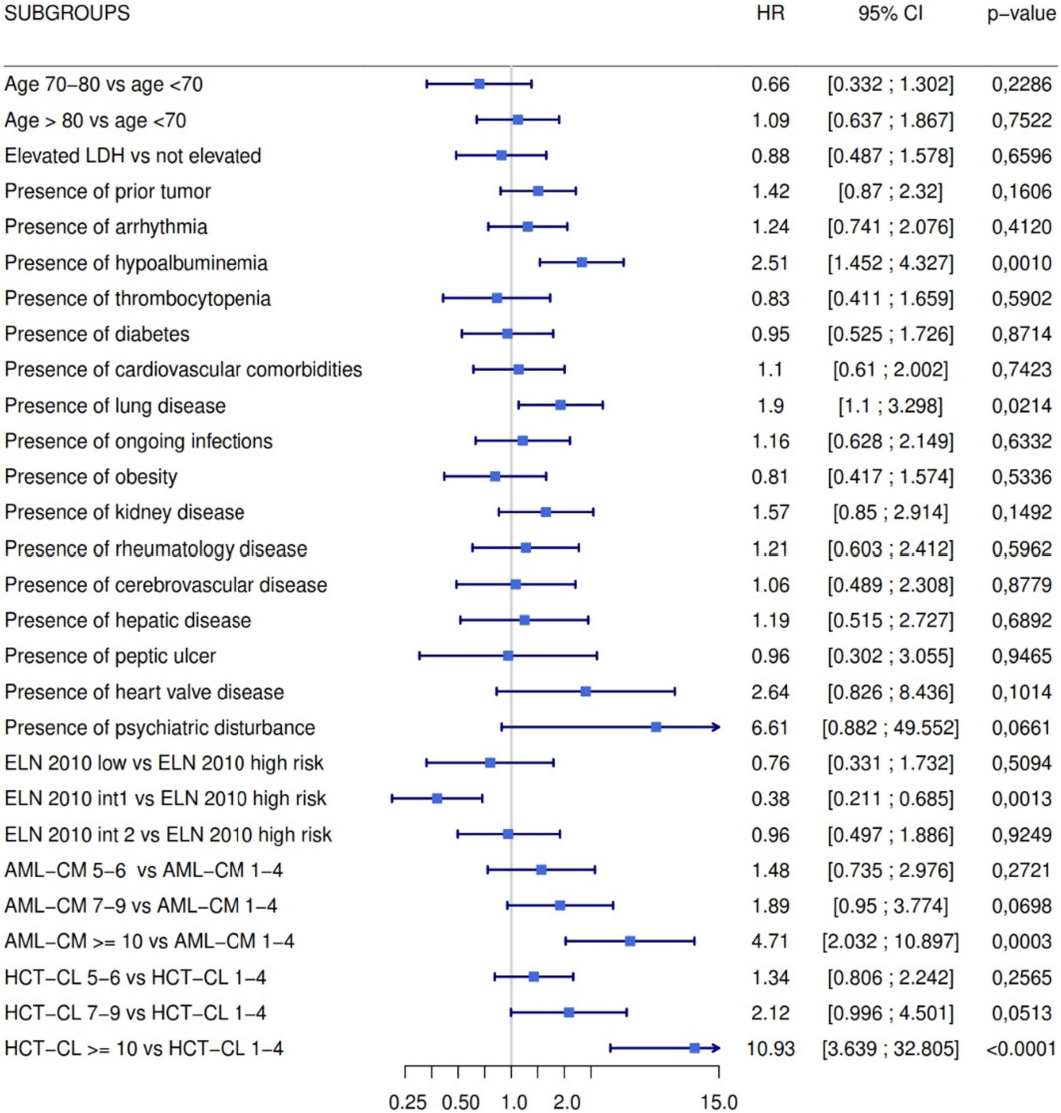
Forest plot for analyzed risk factors.

To test the impact of comorbidities combined with cytogenetic and molecular risk, we used the AML‐CM score. Interestingly, median OS was 24.3 months (95% C.I. 13.9–NR) in patients with low AML‐CM score (1–4, *n* = 24), 18.3 months (95% C.I. 14.2–26.1) in patients with intermediate AML‐CM (5–6, *n* = 45), 11.1 months (95% C.I. 8.0–21) in patients with high AML‐CM score (7–9, *n* = 47) and 6.4 months in patients with very high AML‐CM score (>9, *n* = 15), as shown in Figure 2C (*p* for trend<0.001). Of note, cerebrovascular disease, obesity, lung disease, prior tumor, and hypoalbuminemia were more frequent in patients with AML‐CM score >9 (Table S1). Impact on the prognosis of each baseline comorbidities, age, and scores categories are shown in Figure 3. Taken together, our results indicate that the AML‐CM score was able to stratify prognosis in elderly patients receiving frontline HMAs.

### 3.5 Adverse events have a higher incidence in patients with a higher AML‐CM score

To corroborate and explain the impact of comorbidities on prognosis and survival, we assessed the incidence of adverse events in different AML‐CM risk groups. Particularly, we collected clinically relevant AEs except for hematology toxicity (grade >3). Incidence of non‐hematologic AEs progressively increased along with AML‐CM risk groups. In particular, 84.55, 116.01, 131.45, and 229.3 events for 100 patients per year were observed in patients with AML‐CM scores 1–4, 5–6, 7–9, and >9, respectively. While considering the etiology of AEs, infections were the most represented non‐hematologic adverse events. Of note, infectious episodes were 53.80, 55.10, 85.95, and 140.13 for 100 patients per year for patients with AML‐CM scores 1‐4, 5–6, 7–9, and >9, respectively. Taken together, these data suggest that a higher AML‐CM score due to baseline comorbidities augments the risk to have an adverse event, especially of infective etiology. The impaired prognosis that we observed in AML‐CM higher risk groups may be explained by a higher incidence of infections and their life‐threatening consequences.

## 4 DISCUSSION

To discern how patient‐ and AML‐related factors impact prognosis, we analyzed data from a large typical set of patients who have been assigned to HMAs by physician's choice. Herein, we collected therapy response and applied ELN2010 to describe the prognostic weight of disease‐related factors, and we applied a predefined comorbidity index (i.e., augmented‐CI) to describe patient‐related factors. In this setting, AML‐specific factors alone, as defined by the ELN2010 classification, provided a partial stratification of prognosis, thus indicating that a more comprehensive approach, which takes into consideration both disease‐related biological factors and patient characteristics, may be helpful.

As expected, our set was enriched for secondary diseases and high‐risk karyotype, while core‐binding factor, FLT3 and NPM1 mutated AML were occasional.^26^, ^27^ Our data confirmed that the survival of patients receiving HMAs is significantly influenced by the rate of response.^28^ The response may be considered a measure of the sensitivity of leukemic cells to the therapeutic effect of drugs, being highly associated with leukemia cell‐intrinsic factors such as chromosomal and molecular alterations. In agreement, the ELN2010 high‐risk category had an inferior rate of response. However, when we further analyzed the impact on survival of AML‐related factors, as captured by ELN2010 classification, we observed that their discriminating power was globally weak. Indeed, a scattered picture emerged; “intermediate‐2”, and “high” risk patients have the worst, but similar OS, whereas “intermediate‐1” risk patients had the best OS; as aforementioned, “low” risk patients were under‐represented and should not be considered in this analysis. ELN2010 risk classification is known to be highly prognostic in patients receiving chemotherapy, while its prognostic power in patients receiving less intensive therapies is not fully settled. Notwithstanding, differently from younger intensively treated patients,^29^, ^30^ our data suggest that factors other than AML biology might contribute to define prognosis in elderly patients treated with HMAs.

To evaluate the role of patient‐derived factors as determinants of global clinical outcomes, we focused on baseline comorbidities. We investigated multi‐morbidity taking advantage of the well‐validated augmented‐CI score^20^ and we introduced the concept of comorbidity burden. Most of the patients had at least one comorbidity, as expected in the aging general population.^26^ In particular, the majority of our patients had >1 baseline comorbidity, and >50% had multi‐morbidity. Both hypoalbuminemia, which may be considered a surrogate marker of inflammatory state and poor physical reserve,^29^ and lung disease had a significant impact on prognosis; the other factors had no univariable significance, however, it can be explained by the low incidence in our population. Of utmost importance, having a high comorbidity burden (>6 concomitant comorbidities) seems to be the most impactful determinant of OS. Based on these data, to explain the important impact of comorbidities, we found that a higher baseline comorbidity burden augments the risk to have an adverse event, especially of infective etiology. These results suggest that a higher incidence of adverse events and their life‐threatening consequences may account for the inferior OS we observed in AML‐CM higher risk groups.

Our study has some limitations. It focuses on a cohort of patients receiving only HMAs, thus excluding the relevant impact of new and effective molecules, such as venetoclax and/or target therapies. In our cohort, the overall survival estimate overcomes results previously reported in phase 3 clinical trials of HMAs,^1^, ^2^ probably due to differences in supportive measures such as infections prophylaxis and management. It is important to note that, within a preselected patient set, the age did not confer a significant survival disadvantage, which is partially inconsistent with previously reported results.^21^, ^31^ However, empirical criteria applied by treating physicians may have selected very fit patients, thus providing a selection bias, which may account for the differences in survival in comparison to previous reports. Furthermore, genetic characterization was not integrated into the clinical practice and was not collected for our patients. For this reason, actual or previous ELN risk stratifications^23^, ^32^ cannot be applied; even if this is a limitation for this study, ELN risk stratifications never demonstrated excellent predictive power in the specific population. Finally, a full geriatric assessment was not performed, which may reduce the comparability of our study with previous works based on a geriatric perspective.^13^ Notwithstanding, our study is the largest that analyses the impact of both biology‐ and patient‐related factors, especially baseline comorbidities, in unfit‐for‐chemotherapy AML patients who received HMAs. Indeed, most of the previous reports have focussed on outcomes after intensive chemotherapy^17^ or mixed populations^21^; our study validate AML‐CM in a population that received HMAs as a single agent. In that, our data may be prodromic to understanding the prognostic value of comorbidity in unfit‐for‐chemotherapy elderly patients, undergoing HMAs in combination with novel therapies, such as venetoclax (the study is ongoing). Furthermore, after the validation of patient‐related risk scores, future interventions will be ameliorated with a better selection of the population of patients that may really benefit and by strategies that will moderate the impact of patient‐related risk factors.

In conclusion, the comorbidity burden was a powerful prognostic factor, capable to impact patients' stratification and to integrate a biology‐oriented model. The results of our study further support the notion that baseline multi‐morbidity is tightly related to patient frailty, underlying on‐therapy morbidity, especially of infectious etiology, and contributing to survival. In a scenario where new therapies, such as venetoclax and targeted drugs, have dramatically improved the prognosis of older AML patients, tailored interventions able to impact frailty are warranted. In that, the integration of quantitative assessment of comorbidity burden within a novel prognostic and risk‐classification algorithm, oriented to unfit‐for‐chemotherapy patients, may represent a fundamental step toward a comprehensive approach to elderly AML.

## AUTHOR CONTRIBUTIONS

**Giovanni Marconi:** Conceptualization (equal); investigation (equal); methodology (equal); resources (equal); writing – original draft (lead); writing – review and editing (equal). **Anna Candoni:** Conceptualization (equal); data curation (equal); investigation (equal); resources (equal); writing – original draft (equal); writing – review and editing (equal). **Roberta Di Nicola:** Investigation (equal); resources (equal); writing – original draft (equal); writing – review and editing (equal). **Chiara Sartor:** Resources (equal); writing – original draft (equal); writing – review and editing (equal). **Sarah Parisi:** Resources (equal); writing – original draft (equal); writing – review and editing (equal). **Mariachiara Abbenante:** Resources (equal); writing – original draft (equal); writing – review and editing (equal). **Jacopo Nanni:** Resources (equal); writing – original draft (equal); writing – review and editing (equal). **Gianluca Cristiano:** Resources (equal); writing – original draft (equal); writing – review and editing (equal). **Letizia Zannoni:** Resources (equal); writing – original draft (equal); writing – review and editing (equal). **Davide Lazzarotto:** Resources (equal); writing – original draft (equal); writing – review and editing (equal). **Benedetta Giannini:** Resources (equal); writing – original draft (equal); writing – review and editing (equal). **Carmen Baldazzi:** Resources (equal); writing – original draft (equal); writing – review and editing (equal). **Lorenza Bandini:** Resources (equal); writing – original draft (equal); writing – review and editing (equal). **Emanuela Ottaviani:** Resources (equal); writing – original draft (equal); writing – review and editing (equal). **Nicoletta Testoni:** Resources (equal); writing – original draft (equal); writing – review and editing (equal). **Chiara Di Giovanni Bezzi:** Resources (equal); writing – original draft (equal); writing – review and editing (equal). **Rania Abd‐alatif:** Resources (equal); writing – original draft (equal); writing – review and editing (equal). **Giulia Ciotti:** Resources (equal); writing – original draft (supporting); writing – review and editing (equal). **Renato Fanin:** Resources (equal); supervision (equal); writing – original draft (equal); writing – review and editing (equal). **Giovanni Martinelli:** Resources (equal); writing – original draft (equal); writing – review and editing (equal). **Stefania Paolini:** Resources (equal); writing – original draft (equal); writing – review and editing (equal). **Paolo Ricci:** Resources (equal); writing – review and editing (equal). **Michele Cavo:** Investigation (supporting); project administration (supporting); resources (equal); supervision (equal); writing – original draft (equal); writing – review and editing (equal). **Cristina Papayannidis:** Conceptualization (equal); data curation (equal); investigation (equal); methodology (equal); project administration (lead); resources (equal); supervision (equal); writing – original draft (equal); writing – review and editing (lead). **Antonio Curti:** Conceptualization (equal); data curation (equal); investigation (equal); project administration (lead); resources (equal); supervision (equal); writing – original draft (equal); writing – review and editing (equal).

## CONFLICT OF INTEREST STATEMENT

Dr. Giovanni Marconi received honoraria as consultant/speaker bureau from Menarini/Stemline, Pfizer, Servier, and Astellas; he received research support from Pfizer, AbbVie, and AstraZeneca. Dr. Anna Candoni received honoraria from Pfizer, Amgen, AbbVie, Janssen, Astellas, Celgene. Prof. Giovanni Martinelli acted as a consultant for Ariad/Incite, Jansen, Pfizer, Celgene, Amgen, J&J, and Roche. Prof. Michele Cavo acted as a consultant for and received honoraria from AbbVie, Glaxo Smith Kline, Bristol‐Myers Squib, Adaptive Biotechnologies, Takeda, Janssen, Celgene, Amgen. Moreover, Prof. Cavo is in the speaker's bureau of AbbVie. Dr. Cristina Papayannidis received honoraria from Pfizer, Amgen, Novartis. Dr. Antonio Curti received honoraria from Novartis, Pfizer, Abbvie and acted as a speaker in Advisory Board for Novartis and Abbvie. and Glaxo Smith Kline. The other authors declare no conflict of interest or activities that could appear to have influenced this manuscript.

## Supporting information


Data S1:

## Data Availability

Data will be available upon request at https://www.emato.it/ematogeriatria/
