# Supplementary material for: The baseline comorbidity burden affects survival in elderly patients with acute myeloid leukemia receiving hypomethylating agents: Results from a multicentric clinical study
Source: Cancer Med. 2023 Mar 31;12(10):11838–48. doi: 10.1002/cam4.5858 (PMC10242854; doi:10.1002/cam4.5858)

# Supplementary

Table S1: distribution of baseline comorbidities, age and ELN risk according to AML-CM score risk categories.

| **Variables** | **1≤ AML_CM ≤ 4** | **5≤ AML_CM ≤ 6** | **7≤ AML_CM ≤ 9** | **AML_CM ≥ 10** | **p** |
| --- | --- | --- | --- | --- | --- |
| n | 13 | 25 | 36 | 13 |  |
| Age (median [IQR]) | 77.00 [72.00, 82.00] | 75.00 [72.00, 77.00] | 73.00 [70.00, 78.25] | 76.00 [71.00, 80.00] | 0.352 |
| Age_M75 = >75 (%) | 8 ( 61.5) | 10 ( 40.0) | 14 ( 38.9) | 7 ( 53.8) | 0.464 |
| Age_Cat (%) |  |  |  |  | 0.256 |
| 70≤Age≤80 | 8 ( 61.5) | 19 ( 76.0) | 22 ( 61.1) | 7 ( 53.8) |  |
| Age <70 | 0 ( 0.0) | 3 ( 12.0) | 8 ( 22.2) | 3 ( 23.1) |  |
| Age >80 | 5 ( 38.5) | 3 ( 12.0) | 6 ( 16.7) | 3 ( 23.1) |  |
| ARRYTMIA_POINT_f = Yes (%) | 0 ( 0.0) | 4 ( 16.0) | 12 ( 33.3) | 3 ( 23.1) | 0.062 |
| CARDIOVASCULAR_POINT_f = Yes (%) | 0 ( 0.0) | 1 ( 4.0) | 8 ( 22.2) | 3 ( 23.1) | 0.057 |
| IBD_POINT_f = Yes (%) | 0 ( 0.0) | 1 ( 4.0) | 1 ( 2.8) | 0 ( 0.0) | 1.000 |
| DIABETES_POINT_f = Yes (%) | 1 ( 7.7) | 4 ( 16.0) | 8 ( 22.2) | 2 ( 15.4) | 0.733 |
| CEREBROVASCULAR_POINT_f = Yes (%) | 0 ( 0.0) | 1 ( 4.0) | 5 ( 13.9) | 4 ( 30.8) | 0.048 |
| PSICIATRIC_POINT_f = Yes (%) | 0 ( 0.0) | 0 ( 0.0) | 0 ( 0.0) | 0 ( 0.0) | 1.000 |
| HEPATIC_POINT_f = Yes (%) | 0 ( 0.0) | 1 ( 4.0) | 4 ( 11.1) | 1 ( 7.7) | 0.767 |
| OBESITY_POINT_f = Yes (%) | 0 ( 0.0) | 1 ( 4.0) | 7 ( 19.4) | 4 ( 30.8) | 0.041 |
| INFECTION_POINT_f = Yes (%) | 0 ( 0.0) | 4 ( 16.0) | 7 ( 19.4) | 3 ( 23.1) | 0.361 |
| RHEUMATOLOGICAL_POINT_f = Yes (%) | 0 ( 0.0) | 4 ( 16.0) | 2 ( 5.6) | 2 ( 15.4) | 0.274 |
| PEPTIC_ULCER_POINT_f = Yes (%) | 0 ( 0.0) | 0 ( 0.0) | 2 ( 5.6) | 0 ( 0.0) | 0.759 |
| KIDNEY_POINT_f = Yes (%) | 0 ( 0.0) | 1 ( 4.0) | 8 ( 22.2) | 3 ( 23.1) | 0.057 |
| LUNG_POINT_f = Yes (%) | 0 ( 0.0) | 1 ( 4.0) | 8 ( 22.2) | 6 ( 46.2) | 0.003 |
| PRIOR_TUMOR_POINT_f = Yes (%) | 0 ( 0.0) | 1 ( 4.0) | 9 ( 25.0) | 8 ( 61.5) | <0.001 |
| HEART_VALVE_POINT_f = Yes (%) | 0 ( 0.0) | 0 ( 0.0) | 0 ( 0.0) | 1 ( 7.7) | 0.299 |
| HYPOALBUMINEMIA_POINT_f = Yes (%) | 0 ( 0.0) | 4 ( 16.0) | 8 ( 22.2) | 8 ( 61.5) | 0.002 |
| THROMBOCYTOPENIA_POINT_f = Yes (%) | 2 ( 15.4) | 5 ( 20.0) | 8 ( 22.2) | 5 ( 38.5) | 0.538 |
| LDH_POINT_f = Yes (%) | 10 ( 76.9) | 19 ( 76.0) | 28 ( 77.8) | 12 ( 92.3) | 0.691 |
| AGE_POINT_f = Yes (%) | 13 (100.0) | 25 (100.0) | 36 (100.0) | 13 (100.0) | NA |
| RISK_POINT_ON_ELN2017_f = Yes (%) | 11 ( 84.6) | 22 ( 88.0) | 34 ( 94.4) | 12 ( 92.3) | 0.638 |

Supplementary figure 1: OS for patients in CR/CRi/PR at month 6 and 12


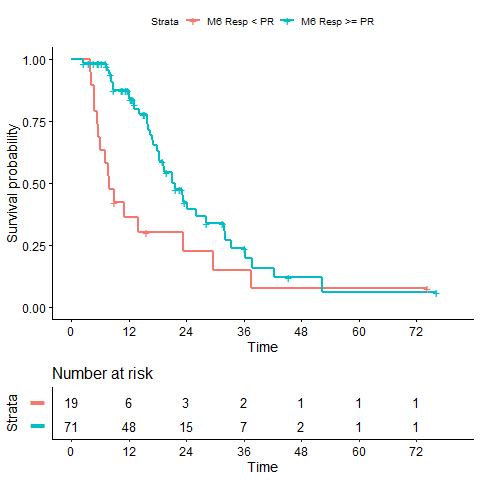

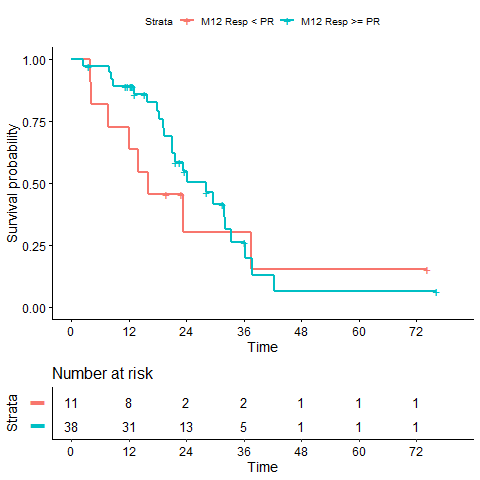

Supplement: Supplementary file 1 — Data S1: [file CAM4-12-11838-s001.docx]
